# Multidisciplinary Management of Complicated Locally Advanced and Fungating Breast Cancer: A Narrative Review

**DOI:** 10.3390/cancers18142254

**Published:** 2026-07-14

**Authors:** Cosmin Vasile Obleagă, Mirela-Marinela Florescu, Lucian Mihai Florescu, Rukie Ana Maria Ahmet, Sergiu Marian Cazacu, Alina Maria Mehedințeanu, Michael Schenker, Liliana Streba

**Affiliations:** 1 Department of Surgical Oncology, University of Medicine and Pharmacy of Craiova, 200349 Craiova, Romania; 2 Department of Pathology, University of Medicine and Pharmacy of Craiova, 200349 Craiova, Romania; 3 Department of Radiology and Medical Imaging, University of Medicine and Pharmacy, 200349 Craiova, Romania; 4 Department of Plastic Surgery, University of Medicine and Pharmacy of Craiova, 200349 Craiova, Romania; 5 Department of Gastroenterology, University of Medicine and Pharmacy of Craiova, 200349 Craiova, Romania; 6 Oncology Department, St. Nectarie Oncology Center, 200349 Craiova, Romania; 7 Department of Oncology, University of Medicine and Pharmacy of Craiova, 200349 Craiova, Romania

**Keywords:** fungating breast cancer, multidisciplinary team, radiotherapy

## Abstract

Context: Locally advanced fungating breast cancer complicated by hemorrhage or infection represents a complex clinical condition that often requires a rapid and coordinated multidisciplinary approach. Although international guidelines, such as those developed by ESMO and NCCN, provide well-defined principles for the management of locally advanced breast cancer, they offer limited recommendations regarding the management of complicated fungating forms. Methods: We conducted a structured narrative review of the literature using the PubMed, Scopus, and Web of Science databases, including studies published between December 2019 and March 2026. Studies addressing locally advanced breast cancer and fungating forms complicated by hemorrhage or infection were included. Results: Available data are heterogeneous and derive primarily from case reports, small case series and retrospective studies. Systemic therapy remains the cornerstone of oncologic control; however, the presence of acute complications often necessitates prioritization of local interventions depending on the patient’s clinical stability. Hemorrhage may be managed through arterial embolization, hemostatic dressings or radiotherapy, whereas infection requires local wound care and targeted antibiotic therapy. These interventions aim to control acute symptoms and stabilize patients, thereby facilitating the subsequent integration of oncologic treatment. Emergency surgical intervention is reserved for selected cases, particularly when conservative measures fail to control bleeding or in the presence of persistent sepsis. Conclusions: The management of fungating breast cancer should be individualized and guided by the clinical presentation within a multidisciplinary team setting rather than by a rigid therapeutic sequence. This paper proposes a pragmatic, symptom-oriented clinical framework integrating all currently available therapeutic modalities (systemic therapy, radiotherapy, interventional radiology, surgery, and supportive care) with the aim of supporting decision-making in complex clinical scenarios.

## Simple Summary

Fungating breast cancer is a rare but challenging clinical condition, frequently associated with ulceration, infection, necrosis, bleeding, malodor, and a significant deterioration in patients’ quality of life. Current international guidelines provide recommendations for the management of locally advanced breast cancer but offer limited guidance regarding these complicated presentations. This narrative review summarizes evidence published between 2019 and 2026 on the multidisciplinary management of fungating breast cancer. Available data are derived primarily from case reports, small case series, and retrospective studies. Systemic therapy remains the cornerstone of oncologic treatment; however, acute complications often require immediate local interventions. Radiotherapy, interventional radiology techniques such as transcatheter arterial embolization, surgery, wound care, and supportive measures may all play important roles depending on the patient’s clinical condition. Based on the available evidence, we propose a pragmatic, symptom-oriented management framework that prioritizes stabilization of acute complications while facilitating the integration of definitive oncologic treatment through multidisciplinary decision-making.

## 1. Introduction

Breast cancer encompasses a broad spectrum of clinical scenarios [1,2]. Despite advances in early detection and improved treatment strategies, it continues to be associated with significant morbidity and mortality rates [2]. Meanwhile, a substantial subset of patients presents for medical evaluation only when an acute progressive complication of breast cancer arises, such as infection or hemorrhage [1,3,4,5,6,7].

Locally advanced breast cancer (LABC) represents a heterogeneous clinical entity corresponding to stage III according to the staging criteria of the American Joint Committee on Cancer [3]. It includes patients with large primary tumors (T3–T4) and/or extensive regional lymph node involvement (N2–N3), in the absence of distant metastases [1,3]. Inflammatory breast cancer constitutes a distinct and clinically and biologically aggressive form classified as T4d. It is characterized by the rapid onset of diffuse erythema, edema (“peau d’orange” appearance) and breast enlargement—the direct macroscopic consequences of the obstruction and widespread engorgement of the dermal lymphatic network by proliferating tumor emboli, usually in the absence of a well-defined tumor mass [1,3]. Inflammatory breast cancer is primarily diagnosed on clinical grounds. The presence of tumor emboli within the dermal lymphatic vessels on skin punch biopsy is a characteristic pathological finding that supports the diagnosis, although their absence does not exclude inflammatory breast cancer when the clinical presentation is typical. Dermal lymphatic invasion is histologically confirmed in only approximately 75% of clinically evident cases.

In this context, distinguishing between LABC, fungating breast cancer and inflammatory breast cancer is essential, as these terms are often used interchangeably despite describing distinct clinical and biological entities. LABC primarily represents a staging category and does not necessarily imply the presence of specific clinical manifestations such as ulceration, infection, or hemorrhage. Fungating breast cancer represents a clinical manifestation of LABC characterized by tumor infiltration and erosion of the overlying skin, leading to extensive ulceration, cutaneous necrosis, exudate and occasionally infection or hemorrhage [1,3,6,8,9,10,11]. These forms are most commonly encountered in the setting of locally advanced breast cancer; however, fungating lesions may also occur in patients with cutaneous metastases. Fungating breast cancer is frequently associated with malodor and wound discharge, factors that significantly impair patients’ quality of life. Although most often encountered in advanced disease and commonly corresponding to T4 tumors, the term “fungating” describes a macroscopic appearance rather than tumor stage or biological behavior [8,9,10,11,12]. In contrast, inflammatory breast cancer is primarily driven by dermal lymphatic invasion rather than necrosis or ulceration and is associated with a poor prognosis [3]. This conceptual distinction has direct implications for therapeutic strategy and treatment sequencing [1,6,9,10,12,13], and the selection of the initial therapeutic modality (chemotherapy, radiotherapy, antibiotic therapy, arterial embolization, surgery or combinations thereof) [1,3,4,5,6,7,8,9,10,11,12,13] must be carefully considered and monitored, as treatment failure may occur.

Contemporary guidelines for advanced breast cancer consistently advocate multidisciplinary decision-making and individualized treatment planning for both inoperable locally advanced and metastatic disease [14,15]. Nevertheless, the literature regarding fungating breast tumors remains fragmented. Consequently, the major unmet need is not another general statement advocating comprehensive care, but rather a practical framework to help clinicians determine which local intervention is appropriate, when it should be applied and how it should be integrated with systemic therapy and supportive care [16,17,18,19,20,21,22,23]. In this context, although international guidelines such as those developed by ESMO [24] and NCCN [25] provide well-structured recommendations for the management of LABC, the approach to acute complications associated with fungating tumors remains insufficiently standardized. For this reason, therapeutic assessment requires integration of LABC-specific oncologic treatment with the management of acute local complications, which frequently influence the sequencing of therapeutic interventions [26,27].

Our review focuses on the multidisciplinary management of local complications such as hemorrhage, infection, necrosis, and tumor wound-related symptoms, as these represent the major immediate therapeutic challenges at first hospital admission, regardless of metastatic status. Under these circumstances, clinicians are confronted with a patient presenting with an acute complication of an oncologic disease that ideally should already have been diagnosed and managed by a multidisciplinary team (MDT), making therapeutic decisions at the initial consultation particularly challenging.

This paper aims to integrate currently available evidence into a pragmatic, symptom-oriented framework to support clinical decision-making in routine practice. Compared with existing reviews, the present analysis incorporates the full spectrum of currently available therapeutic options, including systemic therapy, radiotherapy, surgery, and interventional radiology techniques such as TAE, TACE and TACI, which remain frequently underrepresented in the literature. Furthermore, the inclusion of recent studies (2019–2026) allows a more accurate reflection of current clinical practice and the evolution of therapeutic strategies.

## 2. Materials and Methods

A structured narrative review of the literature was conducted to identify studies addressing the management of fungating breast cancer in the setting of locally advanced disease, including cases complicated by infection or hemorrhage. A structured search was performed in the PubMed, Scopus, and Web of Science databases for studies published between December 2019 and March 2026. Google Scholar was additionally used to identify relevant articles. The search strategy included combinations of keywords and controlled vocabulary terms (including MeSH terms), such as “locally advanced breast cancer”, “fungating breast cancer”, “ulcerated tumor”, “hemorrhage”, “infection”, “radiotherapy”, “interventional radiology” and “wound care.” The core search string combined these concepts as follows: (“locally advanced breast cancer” OR “fungating breast cancer” OR “ulcerated breast tumor”) AND (hemorrhage OR bleeding OR infection OR necrosis) AND (radiotherapy OR embolization OR “interventional radiology” OR surgery OR “wound care”). Corresponding MeSH terms (“Breast Neoplasms”, “Hemorrhage”, “Radiotherapy”, “Embolization, Therapeutic”, and “Wound Healing”) were combined with free-text keywords using the Boolean operators AND/OR and adapted to the specific syntax of each database. Additional studies were identified through manual screening of references from relevant articles.

Case reports and case series, as well as cohort studies and review articles describing clinical management strategies, were included. Studies were excluded for the following predefined reasons: publications in languages other than English (owing to the absence of consistent, verifiable translation); conference abstracts without an available full text; studies not describing the complicated or fungating presentation of the disease; fungating tumors of non-breast origin; and studies reporting overlapping populations or duplicate datasets (to avoid double-counting of cases). Included studies addressed fungating forms of breast cancer in the setting of locally advanced disease, including cases complicated by infection or hemorrhage, and described clinically relevant therapeutic interventions. We also included studies addressing the management of LABC that referenced fungating.

**Figure 1:**
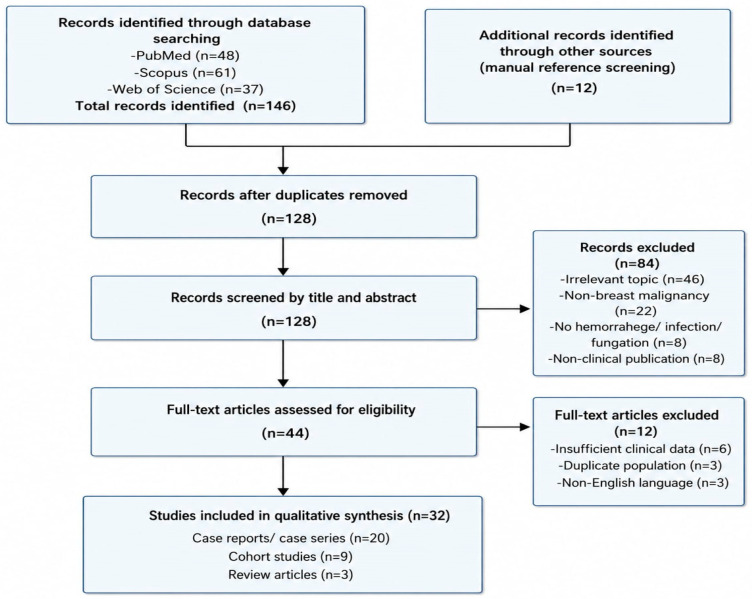
Literature identification and selection process.

To ensure transparency and reproducibility, study selection followed a structured two-stage screening process (Figure 1). After duplicate removal, titles and abstracts were screened, followed by full-text evaluation of relevant articles. A total of 32 studies were included in the narrative synthesis, comprising 20 case reports and case series [1,6,8,9,10,12,21,22,23,24,27,28,29,30,31,32,33,34,35,36], 9 cohort studies [4,11,20,37,38,39,40,41,42], and 3 review articles [43,44,45], which provided information regarding advanced breast cancer complicated by infection or hemorrhage (Table 1). Studies were eligible if they met all of the following explicit criteria: (i) adult patients with locally advanced, fungating, or ulcerated breast cancer complicated by hemorrhage, infection, or necrosis; (ii) description of at least one therapeutic intervention (systemic therapy, radiotherapy, interventional radiology, surgery, or wound care); (iii) study design of case report, case series, cohort study, or review; and (iv) publication in English between December 2019 and March 2026. The present study is a narrative review; a structured search and a two-stage screening process (title/abstract, then full text) were used exclusively to improve the transparency of study selection, without implementation of a systematic review methodology such as formal risk-of-bias assessment or quantitative synthesis. Given the narrative nature of the review and the heterogeneity of the included studies, a formal risk-of-bias assessment was not performed.

## 3. Epidemiology and Clinical Impact

Fungating tumors represent a rare clinical manifestation of advanced breast cancer, with no clearly defined prevalence, and are reported predominantly in the context of delayed presentation [3,36,44]. The incidence of locally advanced breast cancer (LABC) at diagnosis varies substantially across populations and is estimated at approximately 3–5% [3,44,47], while the incidence of complicated locally advanced forms, including fungating tumors, remains relatively low but is associated with a significant clinical burden.

Fungating breast cancer is considered a rare entity in developed countries due to effective screening programs, increased public awareness and easier access to healthcare services, whereas this situation is not similarly observed in resource-limited settings [20]. Factors such as profuse wound discharge, malodor, increased risk of infection, and major psychosocial consequences, including social isolation, contribute substantially to impaired quality of life and further complicate the management of these patients [20]. In this context, comprehensive therapeutic management also requires adequate psychological support.

Nevertheless, analysis of the included studies demonstrated a broad geographical distribution encompassing both developed countries (such as the United States, Japan, Republic of Korea, Italy, and France) and regions with variable healthcare resources (including China, India, Egypt, and Indonesia). The reported age range was broad, spanning from 39 to 90 years, indicating predominant involvement of adult and elderly populations. Although cases were also reported during the COVID-19 pandemic, most included studies originated from the post-pandemic period, suggesting either increased healthcare utilization or more frequent reporting of complex cases in recent years.

Therefore, fungating forms of breast cancer are not confined to specific healthcare systems but rather primarily reflect delayed diagnosis or limited access to medical services. These observations highlight the global nature of this pathology and the need for therapeutic strategies adapted to both clinical and socioeconomic contexts. Once diagnosed, these patients should not be placed on standard waiting lists or subjected to administrative delays but rather managed through expedited pathways. At the same time, such cases should not be approached exclusively from a palliative perspective; a potentially curative intent should also be considered depending on disease stage and tumor biological characteristics [3,4,7].

## 4. Clinical Presentation and Diagnostic Challenges

**Figure 2:**
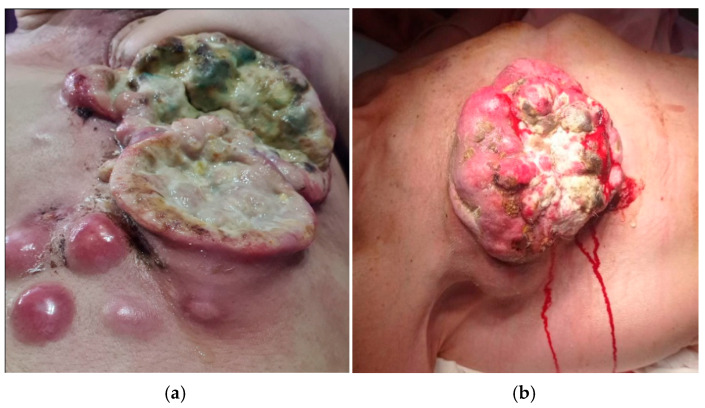
(**a**,**b**) Fungating breast tumors complicated by infection or hemorrhage.

At initial presentation, patients frequently present with ulcerated or fungating breast tumors associated with malodor, active bleeding, or purulent discharge. This clinical picture is more commonly observed in elderly patients, individuals with lower educational levels, or those with significant comorbidities [4,6,20]. Tumor size may vary considerably, ranging from involvement of a single breast quadrant with satellite skin nodules to complete breast involvement with fixation to deeper structures (Figure 2a,b) [46]. The clinical presentation may be further complicated by sepsis, anemia, or metastatic disease. On palpation of the ipsilateral axilla, lymph node masses may be identified either as discrete lesions separate from the primary tumor or as a conglomerate mass [6,9,12].

Although fungating breast tumors are generally associated with advanced disease, the ulcerated and necrotic clinical appearance may also be encountered in other conditions, including severe infections (abscesses, mastitis), inflammatory disorders, or other cutaneous malignancies. In this context, differential diagnosis may be challenging in the absence of additional investigations.

In line with these observations, among the analyzed studies, fifteen reported fungating tumors (including cohort studies [11,20,40,42]), five described hemorrhagic tumors, two reported necrotic lesions, and nine included unresectable locally advanced tumors or cases complicated by infection and/or hemorrhage.

Biological assessment plays an important role in evaluating patients’ overall status and identifying complications associated with advanced disease. The included studies frequently reported anemia secondary to chronic or acute bleeding, as well as elevated inflammatory markers in the setting of local or systemic infection [10,20]. In complicated cases, metabolic disturbances or biological abnormalities suggestive of sepsis may also be identified, potentially influencing therapeutic decisions and requiring stabilization before initiation of disease-specific oncologic treatment [10].

Imaging evaluation plays a crucial role in assessing locoregional extension and distant disease [1,10,12,32]. Among commonly used imaging modalities in breast cancer (ultrasound, computed tomography, and magnetic resonance imaging), computed tomography (CT) is frequently utilized in advanced disease settings, providing relevant information regarding tumor size, invasion of adjacent structures (vascular, osseous, and muscular), and locoregional lymph node status [10,12]. In addition, CT allows identification of distant metastases, with direct implications for oncologic management [10,12]. In emergency settings, CT evaluation may also guide the selection of hemorrhage control strategies (surgical versus interventional), depending on tumor extent and anatomical relationships [10,12,32].

Diagnosis confirmation is established through histopathological examination, usually obtained via biopsy, which allows characterization of tumor type and assessment of key biological markers required for treatment planning [1,32]. In most cases, biopsy is performed before treatment initiation and represents a mandatory step in the diagnostic algorithm [1,32]. However, in emergency situations such as severe hemorrhage refractory to compression and/or embolization [23,28], or in the presence of extensive local infection or uncontrolled sepsis, surgical intervention (if the tumor is technically resectable) may become necessary even before histological confirmation, with diagnosis subsequently established on the surgical specimen [1,32].

## 5. Available Treatment Strategies

### 5.1. Multidisciplinary Assessment and Individualized Therapeutic Planning

The management of complicated locally advanced breast cancer is based on a multidisciplinary approach, consistently supported by the studies analyzed. Given the low level of available evidence—predominantly case reports and small retrospective series—the sequencing suggestions throughout this section should be regarded as pragmatic, expert-opinion guidance rather than evidence-based recommendations. Treatment planning is individualized, taking into account tumor biology, disease extent, patient performance status, and the presence of acute complications such as hemorrhage or infection [3,5].

Individualized therapeutic planning refers to adapting treatment strategy to the specific characteristics of each patient in order to achieve an optimal balance between oncologic control and symptom relief [3,35]. The selection and sequencing of therapeutic interventions are dynamically adjusted according to clinical evolution, with frequent prioritization of acute complication control before initiation of subsequent oncologic treatment [3,5,8,23,28].

Multidisciplinary tumor boards play a central role in establishing therapeutic strategy, ensuring optimal integration between oncologic treatment and symptom management. The multidisciplinary team typically includes a medical oncologist, surgical oncologist, radiation oncologist, radiologist, and pathologist. In complicated cases, the team may be expanded to include an interventional radiologist, plastic surgeon, and infectious disease specialist, particularly in the presence of severe infection or extensive tumor wounds. Wound care specialists, psychologists, intensive care physicians, and palliative care specialists may also be involved to ensure a comprehensive approach adapted to the complexity of the clinical scenario [3,5,9].

In this context, management of these patients requires close collaboration between the surgeon and the medical oncologist, with complementary roles depending on the stage of disease evolution [3,5]. In situations where acute complications such as hemorrhage or infection are controlled, the patient may proceed to systemic oncologic therapy or radiotherapy. Conversely, when these complications persist or worsen, reassessment within the multidisciplinary team becomes necessary, with prioritization of local interventions aimed at clinical stabilization [10,23,28].

Local complications primarily influence short-term clinical evolution and the need for therapeutic interventions, without representing the main determinants of long-term prognosis [4,7,42]. Accordingly, local interventions (surgery, radiotherapy, or interventional procedures) are strategically integrated either as emergency measures, palliative interventions, or conversion strategies aimed at achieving resectability [4,11,34,44].

In standard clinical practice, LABC management follows a well-defined therapeutic sequence that includes comprehensive evaluation and histopathological confirmation, followed by initiation of systemic oncologic treatment (chemotherapy ± radiotherapy), surgery and subsequently tailored adjuvant treatment according to histopathological findings and therapeutic response. This sequential approach aims to optimize locoregional and systemic disease control and is supported by current guidelines [48,49].

However, in fungating tumors complicated by hemorrhage or infection, this therapeutic paradigm is frequently disrupted by clinical urgency. In such situations, although histopathological confirmation remains essential, immediate priority is patient stabilization [32]. Therefore, the optimal approach requires close multidisciplinary coordination in which management of local complications (hemorrhage, infection) and the diagnostic process proceed simultaneously whenever clinical status allows. Management is therefore no longer strictly sequential but becomes simultaneous and adaptive [32].

These observations highlight the need for continuous multidisciplinary reassessment and dynamic adaptation of therapeutic strategy according to tumor response, resectability and clinical evolution.

### 5.2. Systemic Oncological Treatment

Systemic therapy represents the main component of long-term oncologic control in LABC; however, in clinical practice, its initiation may be influenced by the presence of acute complications such as hemorrhage or infection [3,4,44].

Chemotherapy remains the principal therapeutic option in aggressive tumors, particularly in triple-negative breast cancer and HER2-positive disease when combined with targeted therapies [4,11,20,50]. Inflammatory and fungating presentations, in particular, are frequently associated with aggressive molecular subtypes, including HER2-positive and triple-negative disease; consequently, determination of estrogen receptor, progesterone receptor, and HER2 status is essential, as it directly informs the selection of systemic therapy. It is indicated in situations requiring rapid tumor response, such as accelerated disease progression or symptomatic local complications. However, in the presence of severe hemorrhage or sepsis, treatment initiation is generally postponed until clinical stabilization of the patient is achieved [3,10,28]. In the neoadjuvant setting, chemotherapy is administered in sequential cycles over approximately 3–6 months, with periodic reassessment to determine the appropriateness of local treatment interventions [4,8,18]. In advanced disease, treatment is continued until maximal response, disease progression, or significant toxicity occurs [4,11].

Molecular targeted therapy, particularly anti-HER2 treatment, is indicated for patients with HER2-positive tumors and is administered in combination with chemotherapy in most cases [4,18,19]. It contributes to improved tumor response rates and better prognosis. Initiation generally follows the same sequence as chemotherapy. Contraindications are primarily related to treatment-specific toxicities, particularly cardiotoxicity, which requires careful assessment before treatment initiation.

Immunotherapy, particularly immune checkpoint inhibitors (anti-PD-1/PD-L1), is used in selected subgroups of patients with triple-negative breast cancer in combination with chemotherapy [4,19]. Treatment initiation is performed after patient stabilization and exclusion of major contraindications. Treatment duration is variable and is correlated with chemotherapy regimen and therapeutic response. In the presence of severe infection or unstable clinical status, immunotherapy is generally postponed.

In standard LABC management, systemic therapy is frequently administered in the neoadjuvant setting, followed by local treatment and adjuvant therapy [4,18,44,50]. However, in complicated fungating tumors, this sequence is frequently altered, with treatment often initiated through local interventions intended to control acute complications. In the presence of severe local complications, local therapies—including radiotherapy or selective arterial embolization—are prioritized for rapid symptom control, with systemic therapy initiated subsequently [10,23,28]. This approach is supported by analysis of reported cases in which local interventions were frequently used as the first step for patient stabilization [6,7,8,10,21,23,26,27,32,39,40].

Among the analyzed studies, the relationship between histological or molecular tumor subtype and the occurrence of hemorrhagic or infectious complications was not systematically evaluated, and available evidence did not demonstrate a clear correlation between these variables.

The combination of systemic therapy and radiotherapy is feasible in the neoadjuvant setting (neoadjuvant concurrent chemoradiation—NACCRT) [2,4], for palliative purposes, for hemostatic treatment of mild bleeding, or as a conversion strategy to surgery in initially unresectable cases [3,46]. However, in some situations, treatment is administered sequentially in order to limit toxicity. Anti-HER2 therapy may be administered concomitantly with radiotherapy with close monitoring for toxicities, while immunotherapy is combined with radiotherapy only in selected settings and continues to be evaluated in clinical trials [4,19].

Systemic therapy may be safely initiated in the absence of significant post-procedural complications [10,23,28]. In cases of controlled local infection, chemotherapy may be administered concurrently with antibiotic treatment if the patient remains clinically stable. Conversely, in the setting of sepsis, oncologic treatment is temporarily contraindicated, with infection control and patient stabilization taking priority [10,23,28]. Under such circumstances, local interventions may serve as a “bridging therapy,” enabling subsequent initiation of systemic treatment.

The efficacy of systemic chemotherapy in solid tumors is influenced by tumor perfusion and vascular network integrity, factors that may affect distribution of cytotoxic agents [51]. However, the impact of embolization on systemic chemotherapy distribution and efficacy remains poorly defined, and available data in breast cancer are limited. Therefore, embolization should be considered primarily as a stabilization intervention, with systemic therapy initiated after achievement of adequate clinical status.

Regarding chemotherapy resistance, this issue was not systematically analyzed in the included studies, which focused predominantly on symptom control and variability in clinical response without characterization of resistance mechanisms. Nonetheless, situations of tumor progression during treatment have been reported, evidenced by clinical deterioration or the need for changes in therapeutic strategy, although without standardized assessment of this phenomenon [23].

In the included studies, tumor progression or persistent unresectability following neoadjuvant systemic treatment was managed through variable strategies adapted to the clinical context and reassessed within a multidisciplinary setting. Reported approaches included hypofractionated radiotherapy combined with apatinib in difficult-to-control fungating LABC [21], individualized surgical management for progression during neoadjuvant chemotherapy [22], and radiotherapy for downstaging in unresectable or chemotherapy-refractory LABC following neoadjuvant systemic treatment [38].

### 5.3. Radiotherapy

Radiotherapy represents an important component of LABC management, serving both oncologic and palliative purposes depending on disease stage and clinical context [4,9,44]. In fungating tumors, it is used predominantly for local symptom control and patient stabilization and has demonstrated effectiveness in reducing bleeding, inducing tumor regression, and alleviating symptoms such as pain and wound discharge [9,39,40,44].

An important advantage of radiotherapy in this setting is its ability to contribute to control of local complications, including low-intensity tumor hemorrhage, with the hemostatic effect considered to be associated with vascular changes and induction of tumor necrosis [9,38,40,44]. Consequently, radiotherapy may represent a useful option in situations where surgery or interventional procedures are not feasible.

Hypofractionated regimens and palliative protocols are commonly used, allowing achievement of clinical response with an acceptable toxicity profile [35,39,40,44,46]. Radiotherapy can be delivered through various techniques and fractionation schedules, selected according to therapeutic objectives and patient condition [4,44]. In fungating tumors, external beam radiotherapy and palliative or hypofractionated regimens are generally preferred because they allow effective symptom control [35,39,40,44,46]. The “Quad Shot” regimen has shown efficacy in symptom management, particularly in patients with poor performance status or unresectable tumors [46]. Modern techniques such as IMRT and VMAT facilitate improved dose conformity and reduced exposure of healthy tissues [4,38], while emerging approaches may provide additional options for unresectable disease.

Data from case reports and included cohort studies support these observations, with radiotherapy being frequently used in the presence of severe symptoms such as hemorrhage or pain [9,21,39,40,46]. In many cases, radiotherapy enables patient stabilization and continuation of oncologic treatment within a multimodal strategy. However, variability in treatment regimens reflects the lack of standardized protocols, although radiotherapy remains commonly employed in the management of these disease presentations [9,21,39,40,46].

A recent advancement in locoregional control of inoperable fungating tumors is spatially fractionated “lattice” radiotherapy (LRT). In the study by Ferini et al., lattice radiotherapy was used as a local treatment integrated into a multimodal strategy without standardized concurrent systemic therapy [34], and promising results were reported regarding local control and symptom improvement in patients with large ulcerated or fungating breast tumors. This technique involves delivery of higher radiation doses to selected intratumoral regions while sparing surrounding tissues, thereby facilitating favorable clinical responses. Although promising, integration of this approach requires further validation through prospective studies [34].

The use of radiotherapy may be limited by certain clinical conditions: extensive skin lesions, necrosis or severe uncontrolled infection, markedly impaired performance status, or prior irradiation within the same field may require treatment delay or adaptation [40,43]. These situations do not constitute absolute contraindications but rather require careful individualized evaluation. In unresectable infected fungating tumors, radiotherapy may be combined with systemic antibiotic therapy within a multidisciplinary framework after patient stabilization and control of severe infection [9,10].

Given the dynamic evolution of these cases, radiotherapy remains integrated within a multidisciplinary treatment plan and adjusted according to clinical response and the emergence of complications [3,44]. In this context, therapeutic decisions should be reassessed continuously throughout treatment, with radiotherapy potentially facilitating subsequent integration of surgery within a multimodal strategy. A comparative overview of the radiotherapy regimens reported in this setting, including dose–fractionation, local response, symptom relief, and toxicity, is provided in Table 2.

### 5.4. Interventional Radiology

Interventional radiology is part of the therapeutic armamentarium for fungating and complicated breast cancer, providing minimally invasive options for patient stabilization. Arterial embolization was initially used for hemorrhage control in various clinical settings and was subsequently expanded into oncology for management of tumor-related complications. In breast cancer, the use of this technique has been reported mainly in small series and case reports, particularly in the context of fungating tumors complicated by hemorrhage [10,23,26,27]. Through selective occlusion of vessels supplying the tumor, the procedure reduces tumor blood flow and provides prompt bleeding control [10,23,28]. The primary indication for interventional radiology in these cases remains the control of severe or active tumor-related hemorrhage, particularly in emergency settings [10], when conservative treatments are insufficient or contraindicated [10,23,28].

Interventional radiology techniques (Table 3) mainly include transcatheter arterial embolization (TAE), transcatheter arterial chemoembolization (TACE/DEB-TACE), and transcatheter arterial chemoinfusion (TACI). Techniques using drug-eluting beads loaded with chemotherapeutic agents have also been described, with a potential role in local disease control; however, clinical experience remains limited [28,29,31], although these approaches should be considered when breast tumors are unresectable. These minimally invasive procedures allow rapid hemostasis and may serve as a bridge to subsequent oncologic treatment. In the literature analyzed, TAE was the most frequently reported method, whereas chemoembolization was rarely described and generally limited to selected contexts [10,23,26,27].

While TAE may be used as an initial hemostatic intervention, TACE and TACI should preferably be considered only after histopathological confirmation. Selective arterial embolization is performed through an endovascular approach, usually via femoral or radial access, with selective catheterization of arteries supplying the tumor, most commonly branches of the internal mammary artery or lateral thoracic artery. Selective embolization is essential to minimize damage to healthy tissues and reduce the risk of complications [10]. Beyond its hemostatic effect, embolization may also contribute indirectly and to a limited extent to tumor reduction by inducing ischemia and necrosis.

The timing of intervention is critical in managing these patients. In fungating tumors complicated by massive hemorrhage, arterial embolization is frequently used as an initial emergency intervention before initiation of oncologic treatment [24,28]. Data from the literature included in this review suggest that, in selected patients with active hemorrhage and where interventional radiology is available, arterial embolization may be considered an early hemostatic option, as the procedure provides rapid hemostatic effects and enables patient stabilization prior to continuation of oncologic treatment; however, because these data derive predominantly from case reports and small series, this should not be interpreted as a formal first-line recommendation [10,23,26,27].

Radiotherapy and interventional radiology have complementary roles in controlling local complications. Arterial embolization provides rapid hemostatic effects and is particularly indicated for severe active hemorrhage [10,24,26,27], whereas radiotherapy contributes to symptom control and tumor reduction with a more gradual effect and is frequently employed after initial stabilization or in combination with other therapeutic modalities [9,21,39,40]. Local treatment with wound dressings serves primarily a supportive role and is insufficient for controlling significant hemorrhage, being used as an adjunct within multimodal management.

Available cohort studies and review articles do not provide direct comparisons between these modalities, with most data derived from observational series or case reports. Consequently, the effectiveness of each intervention has been reported independently, without standardized comparative analyses. Nevertheless, these findings suggest complementary use of these techniques, with treatment selection determined by the clinical context and urgency of presentation.

Regarding arterial embolization, procedural success may be influenced by factors such as extensive collateral vascularization, difficulties in selective catheterization, or vascular recanalization, all of which may lead to recurrent bleeding. Additionally, in extensively necrotic tumors, diffuse hemorrhagic components may limit procedural efficacy. In general, the technique is well tolerated but may be associated with complications such as post-procedural pain, post-embolization syndrome, infection, or worsening local necrosis. Rare but severe complications may include extensive skin necrosis or non-target embolization [10,28]. Contraindications are mostly relative and include severe uncontrolled infection, uncorrected coagulopathy, or severely impaired general condition, requiring careful risk-benefit assessment before intervention [10,28].

Given the acute nature of these complications and the need for prompt intervention, interventional radiology should be integrated into a multidisciplinary treatment plan and adapted according to the patient’s clinical evolution [3,44].

### 5.5. Surgical Treatment

Surgical treatment remains part of the therapeutic management of breast cancer, including fungating tumors; however, the indication and timing of intervention must be adapted to the particular clinical context of these patients [6,10]. In emergency situations, surgery is reserved for cases in which minimally invasive hemostatic techniques (TAE) are unavailable, ineffective, or when sepsis cannot be controlled [1].

In elective settings, among patients without hemorrhage or with limited bleeding manageable through dressings, as well as those with infected tumors controlled by antibiotic therapy and local wound care, surgical treatment is generally preceded by oncologic therapies [10]. Surgical indications include control of clinically uncontrolled hemorrhage or infection, tumor removal (ideally achieving an R0 resection), and, in selected cases, oncologic control with curative intent, including associated lymphadenectomy. General oncologic literature also supports a possible survival benefit of surgical treatment in selected patients with advanced disease [14,15,52].

In most cases, surgical intervention consists of mastectomy, as breast-conserving procedures are generally not feasible in extensive fungating tumors [6,11,32], even following downstaging. Depending on disease extent, resection may include adjacent structures, and axillary lymphadenectomy is performed when oncologically indicated and technically feasible [7,11].

Although radical Halsted mastectomy no longer represents the standard therapeutic approach in modern breast cancer care, principles of extensive radical surgery may still remain relevant in selected cases of fungating tumors with muscular invasion or significant chest wall extension, particularly when local disease control and achievement of R0 resectability are required [22].

Resectability criteria are essential in patient selection. Extensive chest wall invasion, involvement of vital structures, or major vascular invasion (e.g., axillary vein or neurovascular structures) may limit the feasibility of complete resection and contraindicate surgery with curative intent [7,11,15]. When resection is feasible and oncologically indicated, the goal of surgery is to achieve negative margins (R0). However, intraoperative margin assessment may be limited, and definitive margin status is confirmed postoperatively. According to general oncologic literature, microscopically positive margins (R1) are associated with increased risk of local recurrence [53,54].

Clinical examination and imaging, particularly computed tomography (CT), play an essential role in assessing resectability, especially in emergency settings [6]. Clinical examination may reveal satellite skin nodules that can be incorporated into the resection specimen, while CT allows assessment of local tumor extension, including chest wall, muscular, and osseous invasion, as well as relationships with major vascular structures. CT also provides information regarding axillary lymph node involvement and local complications such as collections or extensive necrosis. Evaluation of systemic disease extent may influence surgical indications, particularly in palliative settings [7,11,52].

In the included studies, no standardized data regarding oncologic resection type (R0 vs. R1) were available, and complete resection was not always achievable due to advanced local extension.

Emergency surgical treatment maintains an important but selective role, being indicated primarily for refractory complications or carefully selected cases with curative potential, while elective surgery may be considered following successful downstaging or downsizing. Similar to other oncologic emergencies, surgery is preceded by patient stabilization, and its role is often palliative, focused on symptom control and quality-of-life improvement, although curative intent may also be possible [55,56].

Available literature does not allow direct comparisons between therapeutic strategies in terms of survival or impact on morbidity, as most data derive from case reports, small series, or heterogeneous retrospective cohorts [6,7,11,20,35,39,41,44]. Nevertheless, surgery contributes significantly to management of these cases and is indicated both for control of acute complications (hemorrhagic or infectious) [1,6,32] and after local stabilization as part of a multimodal strategy [10].

#### Reconstruction, Wound Closure, and Postoperative Considerations

Reconstruction following resection of fungating breast tumors remains particularly challenging because the operative field is often extensive, characterized by ulceration and necrosis, frequently colonized by bacteria, and not infrequently altered by previous radiotherapy [11]. The included studies describe the use of primary wound closure, split-thickness skin grafts, and, in selected cases, local or regional flaps, depending on the size of the defect and the patient’s overall clinical condition [11,41,42].

Whenever oncologically feasible, primary wound closure should be considered because it facilitates wound healing, reduces the need for complex reconstructive procedures, and may promote postoperative recovery and improve patient comfort. However, the feasibility of primary wound closure should never influence the extent of resection, which must adhere to established oncologic principles and prioritize the achievement of negative surgical margins (R0). When primary wound closure cannot be achieved, chest wall defect coverage may require split-thickness skin grafts or coverage with local or regional flaps, depending on the size of the defect, previous radiotherapy, and the patient’s overall clinical condition [11,22,41].

Wound-related complications and postoperative morbidity are more frequent than in standard breast cancer surgery and include wound dehiscence, infection, seroma formation, flap necrosis, and graft loss, particularly in previously irradiated or nutritionally compromised patients [11,22].

Reconstructive planning should be incorporated into the preoperative assessment through a multidisciplinary approach involving the breast surgeon, plastic and reconstructive surgeon, and the multidisciplinary oncology team [22]. The choice of reconstructive technique should be individualized according to the extent of resection, local wound characteristics, previous oncologic treatments, and the patient’s overall clinical condition, with the goals of minimizing postoperative morbidity, achieving satisfactory wound healing, and facilitating the timely resumption of oncologic treatment.

### 5.6. Wound Care and Supportive Management

Wound care and supportive management represent essential components in the treatment of patients with fungating breast cancer, playing a major role in symptom control and quality-of-life improvement. Due to malodor, the psychosocial impact of fungating tumors is substantial, frequently contributing to stigmatization, social isolation, and deterioration of interpersonal relationships. These factors may also influence the hospitalization experience itself.

Ulceration and skin destruction caused by tumor extension lead to loss of skin barrier integrity and exposure of necrotic tissues, promoting bacterial colonization and the development of local infections. These tumors are frequently associated with extensive ulceration, necrosis, profuse discharge, foul odor, pain, and bleeding [36,41,42,43].

Selection of dressings is individualized, with absorbent dressings, hydrocolloids, alginates, and antimicrobial dressings frequently used [41,42,43]. For exudative wounds, absorbent dressings such as alginates and hydrofiber/polyurethane foam dressings are recommended because they facilitate absorption of excess secretions and reduce maceration of perilesional skin [36].

For odor control, topical metronidazole and activated charcoal dressings may be used [36]. Mild hemorrhage may benefit from application of dressings impregnated with epinephrine, silver nitrate, calcium alginate, or absorbable gelatin materials [36]. Local pain management may benefit from topical anesthetics such as 2% lidocaine gel or topical opioids such as morphine hydrogel [36]. Reducing dressing change frequency and using atraumatic dressings may also decrease local pain; however, pain control often requires a multimodal approach including systemic analgesic treatment.

Microbiological data from the analyzed literature are limited, but polymicrobial infections involving aerobic and anaerobic bacteria have been described, including Bacteroides, Porphyromonas, Corynebacterium, and Actinomyces, as well as opportunistic pathogens such as Acinetobacter baumannii in extensive necrotic fungating tumors [12,22]. These findings support the role of barrier disruption and tumor necrosis in infectious complications.

The literature suggests that fungating tumors are frequently polymicrobially colonized, including both aerobic and anaerobic bacteria, with an important role in the development of foul odor [57], although such colonization is not consistently associated with systemic infection. In this context, systemic antibiotic therapy is not routinely indicated and should be reserved for patients presenting clinical signs of systemic infection, extensive cellulitis, or sepsis [41,43].

Obtaining bacterial wound cultures may be useful; however, interpretation of results is limited due to the polymicrobial nature of colonization. In fungating tumors, therapeutic management should not focus exclusively on infection control but also on management of the underlying tumor, since persistence of tumor tissue and local necrosis may promote recurrence of infectious complications.

Supportive management also includes nutritional and psychological support, both of which contribute substantially to improving patients’ quality of life.

## 6. Proposed Therapeutic Management Framework

**Figure 3:**
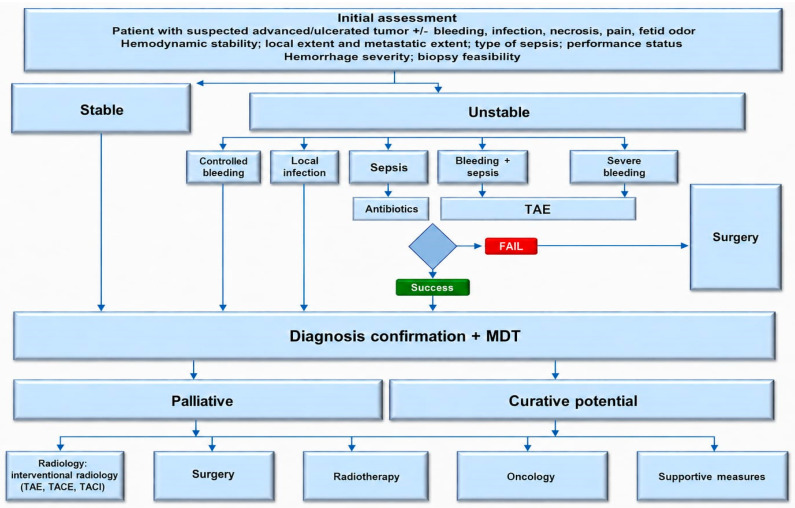
Proposed Pragmatic Multidisciplinary Management Framework for Complicated Locally Advanced and Fungating Breast Cancer. This expert-opinion framework is based on low-level evidence and has not been prospectively validated.

Based on the analyzed data, a pragmatic therapeutic decision model can be proposed in which the initial assessment is directed toward identifying acute complications and evaluating clinical stability, followed by adaptive sequencing of therapeutic interventions (Figure 3). Within this model, control of acute complications represents the first step in clinically unstable cases, followed by initiation of systemic oncologic treatment and integration of local therapies according to clinical response. The framework presented here represents an expert-opinion synthesis derived from low-level evidence (predominantly case reports and small series) and has not been prospectively validated; it is intended to support, rather than replace, individualized multidisciplinary decision-making.

## 7. Future Directions

Recent evolution in therapeutic strategies for complicated LABC reflects a progressive shift toward personalized locoregional approaches aimed at optimizing symptom control and facilitating integration of systemic oncologic treatment.

An important aspect emerging from the analysis of the included studies is that, in the absence of distant metastases, these disease presentations should not be considered exclusively palliative. Within the context of an appropriate multimodal approach and adequate control of acute complications, locally advanced fungating breast cancer may, in carefully selected patients and based predominantly on expert opinion rather than comparative evidence, represent a potentially convertible disease entity that is occasionally amenable to treatment with curative intent. This perspective highlights the importance of proactive therapeutic strategies and avoiding premature classification of such cases as strictly palliative.

Beyond methods already used in clinical practice, such as arterial embolization and radiotherapy, novel locoregional approaches are currently under investigation. Among these, intraductal administration of chemotherapeutic agents has recently been explored in preclinical models, including porcine studies [58], demonstrating feasibility of direct treatment delivery into breast tissue, with increased local distribution and reduced systemic exposure. At present, however, intraductal chemotherapy remains at a preclinical stage, without clinical data in fungating or ulcerated disease, and its potential role is therefore hypothesis-generating rather than immediately translatable to routine practice.

In parallel, advances in radiotherapy, including the development of lattice radiotherapy techniques, reflect a similar trend toward increasing intratumoral dose delivery through spatially fractionated approaches while preserving tolerance of surrounding healthy tissues. Additionally, integration of modern hypofractionated regimens such as the “Quad Shot” protocol reflects the need for rapid and effective interventions for symptom control in patients with fragile clinical status.

These emerging directions suggest convergence of therapeutic strategies toward the concept of targeted locoregional therapy, in which treatment delivery is adapted not only to tumor type but also to route of administration (vascular, intratumoral, or intraductal) [28,37].

Emerging locoregional approaches such as electrochemotherapy (ECT) may also represent additional therapeutic options for selected patients with ulcerated or fungating breast tumors. By increasing intracellular uptake of cytotoxic agents through reversible electroporation, ECT has shown promising results in cutaneous and superficial tumors, including chest wall involvement from breast cancer, particularly with respect to local symptom control and quality-of-life improvement [59,60]. Mechanistically, ECT combines short electric pulses that transiently increase membrane permeability (reversible electroporation) with low-dose cytotoxic agents such as bleomycin or cisplatin, markedly enhancing their intracellular uptake. Evidence in breast cancer derives mainly from cutaneous and chest-wall metastases within registries such as InspECT/ESOPE, where high local response and effective symptom palliation have been reported; nonetheless, data specific to primary fungating breast tumors remain limited, and ECT should currently be regarded as a palliative option for selected superficial lesions.

Modern wound care therapies, including negative pressure wound therapy (NPWT), may also represent promising adjunctive strategies for selected patients with fungating breast cancer, particularly for exudate control and symptom management [61]. However, current evidence remains limited to case reports and small retrospective series, and further prospective studies are needed to clarify indications, safety, and oncologic impact. Malignancy has historically been considered a contraindication to NPWT because of theoretical concerns regarding stimulation of residual tumor growth; however, this absolute contraindication has been questioned, and NPWT is increasingly applied in a carefully selected, palliative manner for exudate control and preparation of the wound bed prior to definitive coverage.

## 8. Limitations

The limitations of this analysis include the heterogeneity of the included studies, the predominance of observational data, and the lack of standardized criteria for evaluating therapeutic response. In addition, hormone receptor and HER2 status was inconsistently reported across the included case reports and small series, which limited subtype-specific conclusions regarding systemic treatment and outcomes. Additionally, the absence of direct comparative studies between different treatment modalities limits the ability to establish a clear hierarchy among available therapeutic approaches.

These limitations highlight the need for dedicated prospective studies addressing this complex clinical setting.

## 9. Conclusions

Locally advanced breast cancer complicated by ulceration, infection, or hemorrhage represents a major clinical challenge and should not be approached as a uniformly palliative entity, but rather as a dynamic oncologic emergency requiring prompt control of acute complications followed by integration of systemic and local oncologic therapies within a multimodal strategy, with the possibility of conversion to resectability in selected cases.

Treatment involves complementary use of systemic therapy, radiotherapy, surgical interventions, and interventional procedures, alongside wound care and supportive management, with the goal of controlling disease and improving quality of life.

Periodic reassessment within a multidisciplinary tumor board is essential for adapting therapeutic strategy according to clinical evolution.

## Figures and Tables

**Table 1:** Chronological summary of included studies (2019–2026). For each included study, the applied treatment and the reported outcome are listed in the “Intervention” and “Outcome/Conclusion” columns, respectively. It should be noted, however, that most of the included case reports and case series were not focused on long-term oncological outcomes, but rather on the management of the acute complication and the subsequent integration into systemic oncological treatment; the level of outcome detail available is therefore necessarily limited.

**Author (Year)**	**Study Type**	**No. of Patients (Age-Median Age)**	**Clinical Setting**	**Intervention**	**Outcome/Conclusion**
Wang et al. (2019) [29] (China)	Case series	15 (mean: 60.8 years)	Unresectable locally advanced breast cancer	Transcatheter arterial chemoembolization (DEB-TACE) using CalliSpheres^®^ microspheres	Feasible locoregional control
Sabir et al. (2020) [6] (Pakistan)	Case report	1 (45 years)	Fungating bleeding tumor	Surgery	Life-saving
Bichoo et al. (2020) [20] (India)	Cohort	79 (mean 55 years)	Fungating tumors	MDT	satisfactory outcome following multimodality therapy; patients receive chemotherapy as the initial component of therapy to enhance their chances of operability.
Yamaguchi et al. (2021) [8] (Japan)	Case report	1 (80 years)	Fungating tumor	MDT	Improving quality of life and tumor control
Chakrabarti et al. (2021) [9] (India)	Case report	1 (50 years)	Bleeding tumor	Palliative Radiotherapy	Symptom control (bleeding, infection, pain)
Liu et al. (2021) [21] (China)	Case report	1 (67 years)	Giant fungating tumor	Radiotherapy + apatinib after postchemotherapy tumor progression	Tumor control
Lin et al. (2021) [31] (Taiwan)	Case report	1 (61 years)	Locally advanced breast cancer	MDT + DEB-TACE +surgery	There was no evidence of local recurrence or distal metastases after 9 months of follow-up.
Ng et al. (2021) [32] (USA)	Case report	1 (90 years)	Fungating tumor	Surgery	Improved quality of life
Jacobson et al. (2021) [39] (Israel)	Cohort	53 (mean 62 years)	Advanced breast cancer including fungating	Palliative radiotherapy	Symptom relief
Hoeltgen et al. (2023) [41] (Germany)	Cohort	26 (mean 60 years)	Advanced breast cancer or local recurrence	Radiotherapy	Palliative RT in symptomatic LABC or locoregional recurrence is an effective treatment option for controlling local symptoms
Poedjomartono et al. (2022) [30] (Indonesia)	Case series	3 (mean 55 years)	Locally advanced breast cancer	Transcatheter arterial chemo infusion (TACI)	Feasible approach
Wei et al. (2023) [28] (China)	Case series	2 (mean 44.5 years)	Ulcerative bleeding tumors	TACE	Effective bleeding control
Abdallah et al. (2023) [11] (Egypt)	Cohort	82 (mean 60.8 years)	Fungating tumors	Surgical algorithm	Effective but associated with morbidity
Wang et al. (2023) [38] (China)	Cohort	71 (mean years)	Locally advanced breast cancer	Radiotherapy	effective tumor downstaging option for chemo-refractory LABC
Kil et al. (2023) [46] (USA)	Case report and review	2 (mean 64)	Neglected breast cancer	Quad-shot radiotherapy	improved QoL and allowed them to receive scheduled SCT without interruption or delay.
Pantelimon et al. (2023) [35] (Romania)	Case report	2 (mean 66.5)	Cancer-related bleeding	MDT-radiotherapy	Tumors response
Atzori et al. (2023) [10] (Italy)	Case report	1 (49 years)	Severe hemorrhage	Embolization	Rapid bleeding control
Osório et al. (2023) [33] (Portugal)	Case report	1 (39 years)	Fungating tumor	Palliative care	Rare pathology
Iyer et al. (2024) [4] (India)	Cohort	202 (mean years)	Inoperable locally advanced breast cancer	neoadjuvant concurrent chemoradiation (NACCRT)	using NACCRT can improve operability and survival outcomes in patients with inoperable LABC
Ferini et al. (2024) [34] (Italy)	Case series	8 (mean 76 years)	LABC fungat	lattice radiotherapy (LRT)	LRT was found to be effective and safe in palliating symptoms among patients with large inoperable breast tumors.
Zhang et al. (2024) [37] (China)	Cohort	30 (mean 55.3 years)	LABC ± fungat	the combination of (DEB-TACE) with systemic chemotherapy	combining systemic chemotherapy with DEB-TACE can translate into long-term progression-free survival (PFS)
Cuniolo et al. (2024) [22] (Italy) Masoudpour et al. (2024) [12] (USA)	Case report Case report	1 (55 years) 1 (51 years)	Progressive locally advanced breast cancer Necrotizing Breast Infection	surgery approach MDT-surgery	importance of a multidisciplinary, tailored treatment approach for each patient with LABC Timely recognition of the condition, the delineation of associated risk factors, and judicious choice of proper management strategies constitute pivotal determinants for optimizing patient prognosis.
Le Tat et al. (2024) [23] (France) Rupert KL et al. (2024) [36] (USA)	Case report Case report	1 (64 years) 1 (51 years)	Massive bleeding Fungating tumors	Arterial Embolization MDT	Bleeding was effectively controlled after embolization and chemotherapy was started. is crucial that more research is conducted to maximize therapeutic interventions, as well as enhance providers’ knowledge of appropriate wound care management.
Reinking et al. (2025) [40]	Cohort	30 (breast) out of 101	Fungating wounds	Radiotherapy	Good response rates
Ganesan et al. (2025) [42]	Cohort	55 (breast) out of 81	Fungating tumors	Supportive care	Improved quality of life
Farzan et al. (2025) [1] (USA)	Case report	1 (71 years)	Necrotizing infection	Surgery	Life-saving intervention
Park et al. (2025) [26] (Republic of Korea)	Case report	1 (67 years)	Resistant triple- negative breast cancer	TAE	TAE has the potential to reduce tumor size and vascularity, making subsequent surgical resection more feasible.
Kang et al. (2025) [27] (Republic of Korea)	Case report	1 (54 years)	Tumor bleeding	TAE	TAE emerges as a viable management strategy for controlling bleeding in palliative care patients with advanced cancer.
Zagardo et al. (2025) [44]	Review	—	Fungating tumors	Multidisciplinary management	Local treatment is crucial for symptomatic palliation
Wong et al. (2025) [43]	Review	—	Wound care	Supportive strategies	Lack of standardization
Firmino et al. (2021) [45]	Review	—	Bleeding malignant wounds	Topical management	Limited evidence

MDT: multidisciplinary Team; TAE: transcatheter Arterial Embolization; TACE: transcatheter arterial chemoembolization; TACI: transcatheter arterial chemoinfusion; QoL: quality of life; DEB-TACE: drug-eluting bead transcatheter arterial chemoembolization. PFS: progression-free survival; NACCRT: neoadjuvant concurrent chemoradiation; LRT: lattice radiotherapy; SCT: systemic Cancer Therapy.

**Table 2:** Comparative overview of radiotherapy regimens reported for fungating and complicated locally advanced breast cancer.

**Regimen/Technique**	**Typical Dose–Fractionation**	**Reported Local Response/Control**	**Symptom Relief (Bleeding, Pain, Discharge)**	**Toxicity Profile**	**Reference(s)**
Conventional palliative EBRT	30 Gy/10 fx or 20 Gy/5 fx	Symptomatic response in the majority of treated patients	Effective control of bleeding, pain and exudate	Generally mild acute skin toxicity	[38,39,41]
Hypofractionated EBRT	30–36 Gy in weekly fractions	Partial regression; durable local control in selected cases	Rapid palliation of bleeding and pain	Acceptable; manageable skin reactions	[21,39,40]
“Quad Shot”	3.7 Gy × 4 over 2 days, up to 3 cycles	Meaningful palliative response without interrupting systemic therapy	Good control in poor-performance-status patients	Low toxicity; well tolerated	[46]
Lattice/spatially fractionated RT (LRT)	High-dose intratumoral vertices with peripheral sparing	Promising local control in large ulcerated/fungating tumors	Symptom improvement reported	Limited data; favorable early profile	[34]

EBRT: external beam radiotherapy; fx: fractions; Gy: Gray; LRT: lattice radiotherapy.

**Table 3:** Overview of interventional radiology techniques in the management of hemorrhagic and fungating locally advanced breast cancer.

**Technique**	**Chemotherapy Delivery**	**Embolization**	**Typical Clinical Use**	**Main Therapeutic Goal**
TAE	No	Yes	Acute tumor bleeding/hemostatic control	Hemostasis
TACI	Yes	No	Selected locoregional therapy	Locoregional drug delivery
TACE	Yes	Yes	Hypervascular or unresectable tumors with bleeding risk	Tumor control and hemostasis

TAE: Transcatheter Arterial Embolization; TACI: Transcatheter Arterial Chemoinfusion; TACE: Transcatheter Arterial Chemoembolization.

## Data Availability

The data presented in this study are available on request from the corresponding author.
